# Editorial: CSF and blood biomarkers in COVID-19 and other neuroinfectious diseases

**DOI:** 10.3389/fneur.2023.1239750

**Published:** 2023-07-11

**Authors:** Matteo Foschi, Samir Abu-Rumeileh, Federico Massa, Christian Cordano, Ahmed Abdelhak

**Affiliations:** ^1^Department of Neuroscience, Neurology Unit, S. Maria delle Croci Hospital, AUSL Romagna, Ravenna, Italy; ^2^Department of Biotechnological and Applied Clinical Sciences, University of L'Aquila, L'Aquila, Italy; ^3^Department of Neurology, Martin-Luther-University Halle-Wittenberg, Halle (Saale), Germany; ^4^Department of Neuroscience, Rehabilitation, Ophthalmology, Genetics, Maternal and Child Health, IRCCS Policlinico San Martino, University of Genova, Genoa, Italy; ^5^Department of Neurology, University of California, San Francisco (UCSF), San Francisco, CA, United States

**Keywords:** COVID-19, biomarker, diagnosis, prognosis, neuroinfectiology

The past decades have witnessed advances in the development of ultrasensitive assays to measure biofluid markers to improve the diagnosis and characterization of central nervous system (CNS) involvement in primary and non-primary neurological diseases (1, 2). Neuroinfectious diseases encompass a broad spectrum of conditions where CNS injury may result from direct pathogen invasion and systemic or compartmentalized inflammatory damage. The recent outbreak of the COVID-19 pandemic has boosted a growing interest in the field of biomarker application in neuroinfectious diseases. Indeed, several neurological manifestations have been described in association with COVID-19, spanning from general symptoms (i.e., delirium, headache, dizziness, and anosmia) to CNS or peripheral nervous system (PNS) syndromes (i.e., encephalopathy, encephalitis, stroke, and Guillain-Barré syndrome spectrum) diseases (1). The investigation of novel biofluid markers reflecting CNS/PNS injury is of clinical relevance given the evidence of a worse prognosis not only in patients with COVID-19-related major neurological manifestations (3) but even in those with asymptomatic/paucisymptomatic neuro-axonal injury (4).

Among all available biomarkers, neurofilament light chain protein (NfL) has been extensively studied as a biomarker of neuro-axonal injury and showed a multifaceted profile of clinical applicability, going beyond prognostication of primary neurological conditions (1). Nevertheless, blood NfL concentration measured with highly sensitive assays may be influenced by several physiological or paraclinical variables, including age, renal function, body mass index (BMI), and the biological matrix of the sample (EDTA-plasma or serum) (1). The latter was a significant hurdle that hampered using NfL in clinical practice and population-based epidemiological studies. Rübsamen et al. investigated potential solutions for a better comparability of NfL concentrations measured in distinct biological matrices to harmonize findings across epidemiological studies. The authors confirmed the numerical differences between EDTA-plasma and serum NfL levels and derived a formula for converting NfL values from EDTA-plasma to serum, so that reference values for NfL standardization [i.e., the recently introduced Z scores (5)] can also be applied if only EDTA-plasma NfL levels are available. This formula represents an exciting step forward for broader applications of NfL in different clinical and research settings.

The role of NfL was highlighted in another study of the present issue. Havdal et al. investigated the relationship between neurological symptoms, cognitive performance (verbal and auditory working memory, verbal learning, delayed recall, and recognition), and blood biomarkers of brain injury (NfL and glial fibrillary acidic protein-GFAP) in a cohort of 405 adolescents and young adults (12–25 years) with paucisymptomatic COVID-19. They reported slightly higher levels of serum NfL and GFAP, while cognitive performance and clinical neurological examination did not differ compared to age-matched non-COVID-19 controls. Neurological symptoms such as fatigue, subjective cognitive difficulties, sleep alterations, and pain were not associated with increased serum NfL and GFAP levels nor with cognitive test results but showed a strong correlation with female sex and older age. These findings suggest a potential disconnection between self-reported neurological symptoms and biological abnormalities in mild COVID-19. Therefore, the authors argued that persisting clinical disturbances in these patients might rely more on biopsychological factors than ongoing underlying structural CNS damage.

The potential of CSF biomarkers goes beyond NfL and GFAP. Indeed, the study by Huang et al. used an unsupervised hierarchical clustering analysis to outline a disease-specific CSF protein profile to improve the diagnosis of tuberculous meningitis (TBM). Specifically, a combination of 3 biomarkers (APOE, APOAI, S100A8) yielded the highest ability in discriminating TBM patients from cases with non-tubercular meningitis with an area under the curve (AUC) of 0.916 (95% sensitivity and 77.5% specificity). The higher expression of APOE proteins may reflect a dysregulation in CNS lipids metabolism directly caused by the pathogen. Whilst APOE proteins are mainly released by astrocytes, microglia, and neurons (6), S100A8 is a proinflammatory mediator primarily expressed by neutrophils and monocytes and involved in the maintenance of chronic inflammation. Of note, a model incorporating biomarkers related to different pathophysiological mechanisms reached the highest diagnostic accuracy, thus suggesting a complex interplay of intrinsic CNS and pathogen-induced immune activation in TBM.

Similarly, the study by Zhao et al. investigated a broad panel of CSF biomarkers to identify potential diagnostic tools for post-neurosurgical bacterial meningitis (PSBM) in the CSF of 31 patients with aneurysmal subarachnoid hemorrhage (aSAH) using Olink^®^ proteomic platform. A total of 15 proteins were upregulated in patients with PSBM compared to those without, and three proteins (PTN, CD27, ANGPT1) yielded the highest diagnostic accuracy, thus outlining a potential distinct immunological profile of PSBM.

Finally, the study by He et al. evaluated the prognostic value of cellular elements evaluated in standard CSF cytology, particularly blood neutrophil-to-lymphocyte ratio (NTR) and monocyte-to-lymphocyte ratio (MLR) in patients with viral encephalitis (VE). The authors found significantly higher NTR and MLR at hospital admission in patients with poor (modified Rankin scale–mRS-score ≥ 2) vs. good (mRS ≤ 1) prognosis at 12-month follow-up. Additionally, increased NTR and MLR were identified as independent predictors of 12-month poor functional outcomes. These findings might improve the early identification of critically ill VE patients who might benefit from more intensive monitoring and early treatment escalation to prevent poor prognosis.

## Conclusion

**Figure 1:**
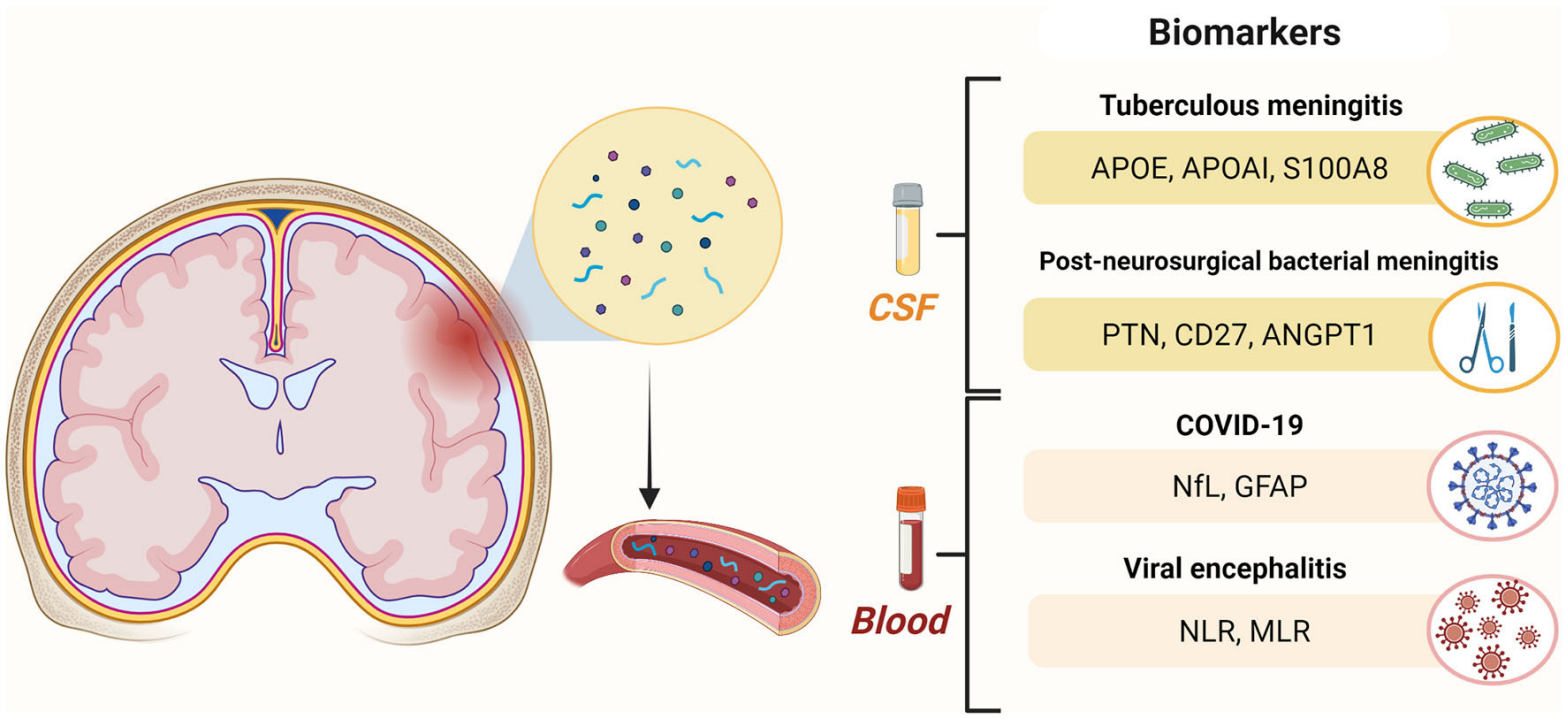
Cerebrospinal fluid (CSF) and blood biomarkers of neuroinfectious diseases investigated in studies included in the Research Topic. ANGPT1, angiopoietin 1; APOAI, apolipoprotein AI; APOE, apolipoprotein E; GFAP, glial fibrillary acidic protein; NfL, neurofilament light chain protein; MLR, monocyte-to-lymphocyte ratio; NLR, neutrophil-to-lymphocyte ratio; PTN, pleiotrophin; S100A8, s100 calcium binding protein A8. Created with BioRender.com.

In conclusion, all the articles included in this Research Topic provide relevant advancements to implement CSF and blood markers in the diagnostic and prognostic assessment COVID-19 and other neuroinfectious diseases (Figure 1) and offer novel insights into underlying pathophysiological processes. Furthermore, the study by Rübsamen et al. provides a possible solution to harmonize NfL levels when obtained in different biological matrices, thus potentially representing a significant step forward in the long road toward clinical validation.

## Author contributions

All authors listed have made a substantial, direct, and intellectual contribution to the work and approved it for publication.
